# The Clinical Impact of Cerebellar Grey Matter Pathology in Multiple Sclerosis

**DOI:** 10.1371/journal.pone.0096193

**Published:** 2014-05-02

**Authors:** Alfredo Damasceno, Benito Pereira Damasceno, Fernando Cendes

**Affiliations:** Department of Neurology, University of Campinas (UNICAMP), Campinas, Brazil; Institute Biomedical Research August Pi Sunyer (IDIBAPS) - Hospital Clinic of Barcelona, Spain

## Abstract

**Background:**

The cerebellum is an important site for cortical demyelination in multiple sclerosis, but the functional significance of this finding is not fully understood.

**Objective:**

To evaluate the clinical and cognitive impact of cerebellar grey-matter pathology in multiple sclerosis patients.

**Methods:**

Forty-two relapsing-remitting multiple sclerosis patients and 30 controls underwent clinical assessment including the Multiple Sclerosis Functional Composite, Expanded Disability Status Scale (EDSS) and cerebellar functional system (FS) score, and cognitive evaluation, including the Paced Auditory Serial Addition Test (PASAT) and the Symbol-Digit Modalities Test (SDMT). Magnetic resonance imaging was performed with a 3T scanner and variables of interest were: brain white-matter and cortical lesion load, cerebellar intracortical and leukocortical lesion volumes, and brain cortical and cerebellar white-matter and grey-matter volumes.

**Results:**

After multivariate analysis high burden of cerebellar intracortical lesions was the only predictor for the EDSS (p<0.001), cerebellar FS (p = 0.002), arm function (p = 0.049), and for leg function (p<0.001). Patients with high burden of cerebellar leukocortical lesions had lower PASAT scores (p = 0.013), while patients with greater volumes of cerebellar intracortical lesions had worse SDMT scores (p = 0.015).

**Conclusions:**

Cerebellar grey-matter pathology is widely present and contributes to clinical dysfunction in relapsing-remitting multiple sclerosis patients, independently of brain grey-matter damage.

## Introduction

In recent years, several neuroimaging studies have shown diffuse grey matter (GM) damage in multiple sclerosis (MS) patients, involving both cortical and subcortical structures, such as the spinal cord and cerebellum. Indeed, modern neuropathological research confirmed these findings and proposed the cerebellum as an important location for cortical demyelination in MS, particularly in those with primary or secondary progressive disease.[1]–[3] Similar types of cortical lesions, as described in the forebrain, are also seen in the cerebellum, such as intracortical and leukocortical. These lesions are also characterized by complete demyelination with relative preservation of neurons and axons.[1], [3] However, the functional significance of these lesions is not completely clear. Moreover, besides its key role in motor function, increasing evidence supports a significant function of the cerebellum in cognition, dependent upon the existence of different anatomical connections between high-level cortical regions, which may also be involved in MS lesions.[3]–[5] Although there are some studies reporting association between MRI atrophy measures and clinical performance, very few evaluated the clinical and cognitive impact of these cortical lesions.[6]–[8] Therefore, in this study, we evaluated the influence of cerebellar GM pathology, as measured by MRI, in clinical and cognitive functions in a group of patients with relapsing remitting MS, addressing relative contributions of cerebellar cortical and white-matter (WM) atrophy, and also cerebellar leukocortical and intracortical lesions.

## Methods

### Subjects

We prospectively and consecutively enrolled 42 patients with a relapsing-remitting MS diagnosis according to the revised 2005 McDonald criteria,[9] and 30 age- and gender-matched healthy control subjects for comparison as a control group. All individuals were evaluated at the MS Center of UNICAMP University Hospital, Campinas, Brazil. All patients were clinically stable (no relapse in the previous three months), with age ranging from 20 to 50 years-old, and on treatment with disease-modifying drugs (Table 1). Exclusion criteria were: progressive course, fulfillment of diagnostic criteria for neuromyelitis optica, EDSS >5.0, any pre-existing condition known to be associated with brain atrophy or any relapse or steroid therapy within three months preceding the clinical and MRI evaluation.

**Table 1:** Clinical and MRI data.

	RRMS patients	Controls	Between-Subjects Comparisons *
Sex: no. (%) Female/Male	32 (76.2)/10 (23.8)	23 (76.7)/7 (23.3)	0.596
Age: years	30.52±6.60	29.52±7.52	0.352
Education: years	13.69±1.83	15.18±0.77	0.001
Disease duration: years	6.40±4.94	NA	NA
Treatment: no. patients (%) IM IFN β-1a/SC IFN β-1b/SC IFN β-1a/SC GA	14 (18.4)/15 (19.7)/7 (9.2)/6 (7.9)	NA	NA
EDSS score	2.5 (0 – 4)	NA	NA
FS cerebellar	0 (0 – 3)	NA	NA
9HPT: seconds	22.92±4.43	17.83±1.49	F = 17.24; p<0.001
T25FW: seconds	4.80±0.85	4.05±0.67	F = 7.94; p = 0.001
PASAT: raw score	33.02±13.01	43.66±9.44	F = 13.20; p<0.001
SDMT: raw score	50.38 ± 13.31	65.14±11.37	F = 16.75; p<0.001
Cerebellar GM volume: cm3	92.77±12.92	94.37±±11.43	F = 15.63; p<0.001
Cerebellar WM volume: cm3	27.37 ± 4.71	29.03±3.52	F = 3.45; p = 0.021
Brain cortical volume: cm3	432.19±41.52	440.83±35.14	F = 9.27; p<0.001
Cerebellar intracortical lesions: cm3	0.05±0.08	NA	NA
Cerebellar intracortical lesions: no. (range)	0.54±0.91 (0–4)	NA	NA
Cerebellar leukocortical lesions: cm3	0.05±0.10	NA	NA
Cerebellar leukocortical lesions: no. (range)	0.69±1.00 (0–4)	NA	NA
Brain WM lesions volume: cm3	6.19±9.19	NA	NA
Brain cortical lesions: cm3	0.85±0.87	NA	NA

Expressed are mean values and standard deviation, except for EDSS and FS cerebellar scores, where a median and range are provided.

9HPT: nine-hole peg test; EDSS: Expanded Disability Status Scale; FS: functional system; GA: glatiramer acetate; GM: grey-matter; IFN: interferon; IM: intramuscular; NA: not applicable; PASAT: Paced Auditory Serial Addition Test; RRMS: relapsing-remitting multiple sclerosis; SC: subcutaneous; SDMT: Symbol Digit Modalities Test; T25FW: timed twenty-five foot walk test; WM: white-matter.

* Group comparisons were performed with Mann-Whitney *U* tests for age and education; Fisher's exact test for gender distribution; and the General Linear Model with gender and total intracranial volume as covariates for volumes comparisons and with education as covariate for clinical tests performances.

### Ethics Statement

The study was approved by the ethics committee of the faculty of medical sciences of University of Campinas and all individuals provided written informed consent.

### Clinical assessment/Outcome measures

Neurological clinical examination included: assessment of leg function by means of the timed twenty-five foot walk (T25FW) and arm function with the nine-hole peg test (9HPT) for all participants. We also measured overall disability with the Expanded Disability Status Scale (EDSS) for all patients and the cerebellar Functional System (FS) score.[10] Cognitive evaluation included the Paced Auditory Serial Addition Test (PASAT) and Symbol Digit Modalities Test (SDMT) for all individuals. Each of these tests was considered different outcome measures.

### Magnetic resonance imaging

MRI scans for all subjects were acquired on a 3T scanner (Phillips Achieva-Intera). The study protocol consisted of: fluid attenuated inversion recovery (FLAIR) – acquired in the axial plane with 3 mm slice thickness (TR 11000 ms, TI 2800 ms, TE 125 ms, matrix 328×210, gap 0, FOV 23×18 cm, flip angle 90°; in-plane resolution 0.7 mm×0.85 mm); double inversion recovery (DIR) - acquired in the axial plane with 3 mm slice thickness (TR 11000 ms, TI 3400 ms, TE 50 ms, delay 325 ms, matrix 328×210, gap 3, FOV 23×18 cm, flip angle 90°; in-plane resolution 0.7 mm×0.85 mm) and a volumetric (three-dimensional) T1 gradient echo images - acquired in the sagittal plane with 1 mm slice thickness (TR 7.0 ms, TE 3.2 ms, matrix 240 × 240, FOV 24 × 24 cm, flip angle 8°; in-plane resolution 1.0 mm×1.0 mm).

### Image Analysis

#### Brain – WM

Brain WM lesion load (WML) was quantified on FLAIR sequences, blinded to clinical data, using the freely available Medical Image Processing, Analysis, and Visualization (MIPAV) software package developed at the Center for Information Technology, National Institutes of Health.[11] The intrarater reliability between brain WML quantification was assessed using intra-class correlation (with three sets of data points; ICC = 0.972); Infratentorial lesions were not included in brain WML.

#### Brain – GM

Brain cortical lesions were identified and scored on DIR sequences, blinded to clinical data, in accordance to consensus recommendation, [12] and accurately controlled for artifacts. Lesion volume was quantified using the MIPAV software. The intrarater reliability between lesion quantification was assessed using intra-class correlation (with three sets of data points; ICC = 0.896). Brain cortical volume evaluation was performed on volumetric T1 gradient echo images by means of the FreeSurfer v5.1 image analysis suite, available online (http://surfer.nmr.mgh.harvard.edu/) (left + right hemispheres volumes), as described elsewhere.[13]–[15] All images were systematically controlled for errors and artifacts.

#### Cerebellum – WM

Presence of cerebellar WM lesions was analyzed on both FLAIR and DIR sequences. Cerebellar WM volume evaluation was performed on volumetric T1 gradient echo images by means of the FreeSurfer v5.1 image analysis suite, available online (http://surfer.nmr.mgh.harvard.edu/) (left + right hemispheres volumes), as described elsewhere.[13]–[15] All images were systematically controlled for errors and artifacts.

#### Cerebellum – GM

**Figure 1:**
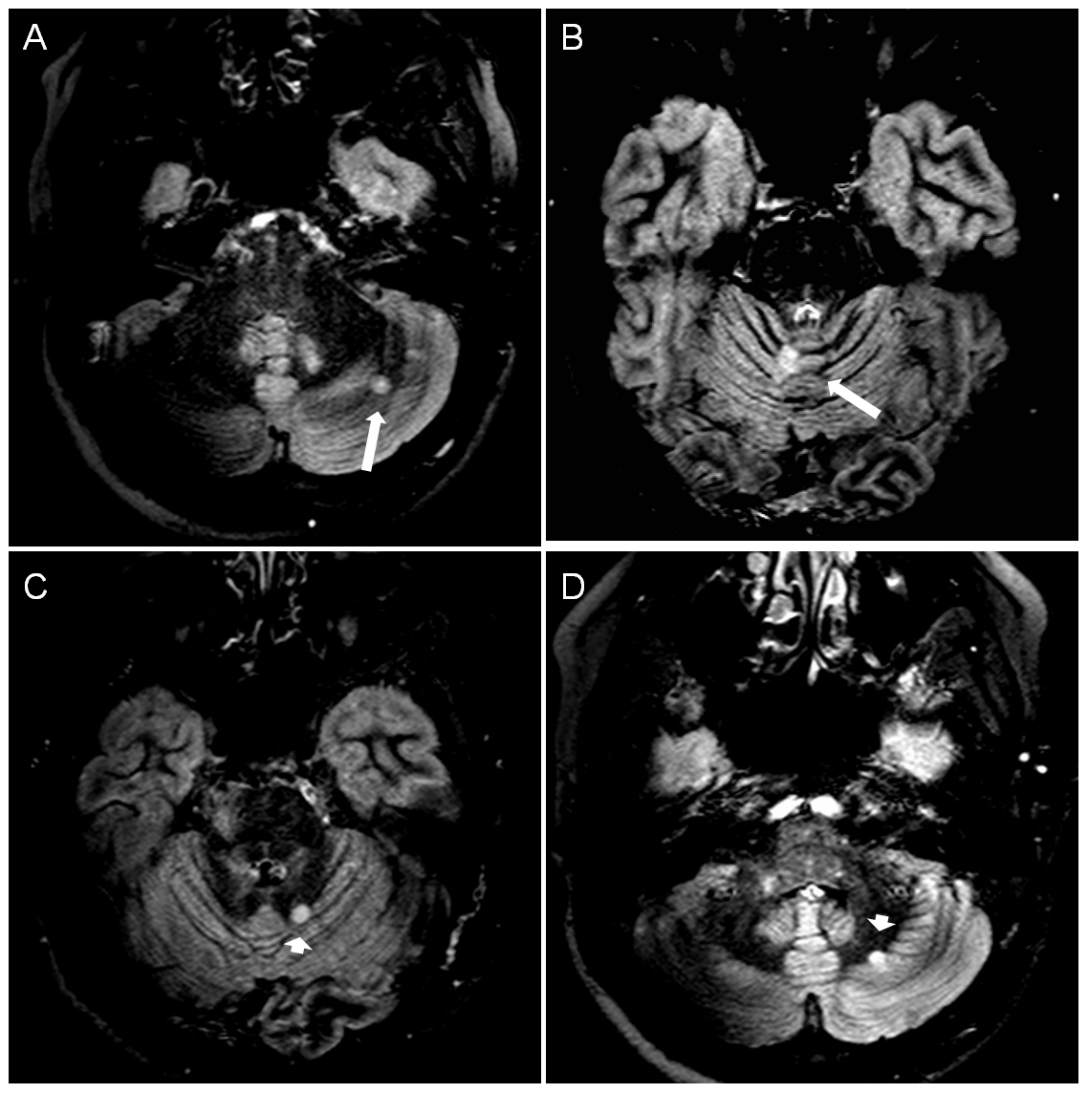
Cerebellar intracortical and leukocortical lesions. Axial double inversion recovery images from relapsing-remitting MS patients showing intracortical (long arrows, A and B) and leukocortical lesions (short arrows, C and D).

Cerebellar GM lesions were identified and scored on DIR sequences, blinded to clinical data, in accordance to consensus recommendation,[12] and accurately controlled for artifacts. Lesion volume was quantified using the MIPAV software. Cerebellar GM lesions were further classified as being intracortical or leukocortical (Figure 1):

Intracortical - when lesion borders remained completely within the cortex.

Leukocortical - when lesions involved the WM/GM junction.

The intrarater reliability between lesion quantification was assessed using intra-class correlation (with three sets of data points; ICC = 0.949 for both);

Cerebellar GM volume evaluation was performed on volumetric T1 gradient echo images by means of the FreeSurfer v5.1 image analysis suite, available online (http://surfer.nmr.mgh.harvard.edu/) (left + right hemispheres volumes), as described elsewhere.[13]–[15] All images were systematically controlled for errors and artifacts.

### Statistical analysis

Statistical analysis was performed using the Statistical Package for the Social Sciences (SPSS, Version 20.0., SPSS Inc, Chicago, Illinois).

Comparisons on brain and cerebellar volumes were analyzed by the General Linear Model (GLM) univariate analyses of variance procedure, with gender and total intracranial volume as covariates. Group comparisons on clinical and cognitive tests were also performed with GLM with number of years of education as covariate.

Spearman correlation analyses were performed to test the associations between clinical and MRI factors and clinical/cognitive outcomes. We also performed partial correlations of cerebellar cortical lesions, EDSS, and cerebellar FS score controlling for brain cortical lesions and brain cortical volume (as covariates).

A stepwise multivariate linear regression analysis was performed initially to assess a possible relative contribution of demographic variables (age, disease duration, years of education and gender) in clinical (EDSS, cerebellar FS, 9HPT and T25FW) and cognitive outcomes (PASAT and SDMT) and then, the same procedure was used to assess the relative contribution of MRI factors (cerebellar intracortical and leukocortical lesions, brain WML, brain cortical lesions, brain cortical volume, and cerebellar WM and GM volumes) in the same clinical and cognitive outcomes. Given a non normal distribution of cerebellar and brain lesion volumes, these variables were dichotomized to low/high burden of lesions based on the mean value (cerebellar intracortical and leukocortical lesions ≥ 0.05 cm3; brain cortical lesions ≥ 1 cm3; and brain WML ≥ 3 cm3).

We also assessed the relative contribution of disease duration, age, and MRI lesions (brain and cerebellum) to cerebellar GM and WM volume. Backward stepwise analyses were conducted (Wald statistic) with a p value for entry of 0.05 and a p value for removal of 0.1. The level of significance was p<0.05.

## Results

### Clinical and MRI characteristics

Clinical and MRI features are shown in Table 1. Cerebellar intracortical and/or leukocortical lesions were observed in 53.8% of the patients (leukocortical in 43.6% and intracortical in 33.3%). Cerebellar WM lesions were found in 62.5% of the patients (Overall, 70.7% of the patients had cerebellar WM and/or GM lesions).

Patients and controls were similar regarding age and gender distribution, but had different years of education. Therefore, group comparisons on clinical and cognitive tests were also performed with number of years of education as covariate. There was no difference regarding gender or presence of oligoclonal bands on performances of clinical/cognitive tests or volumes of brain/cerebellar lesions, except for the T25FW test, where women took longer than men (4.99 vs. 4.20 seconds, p = 0.011).

### Outcome measures

#### Clinical

**Figure 2:**
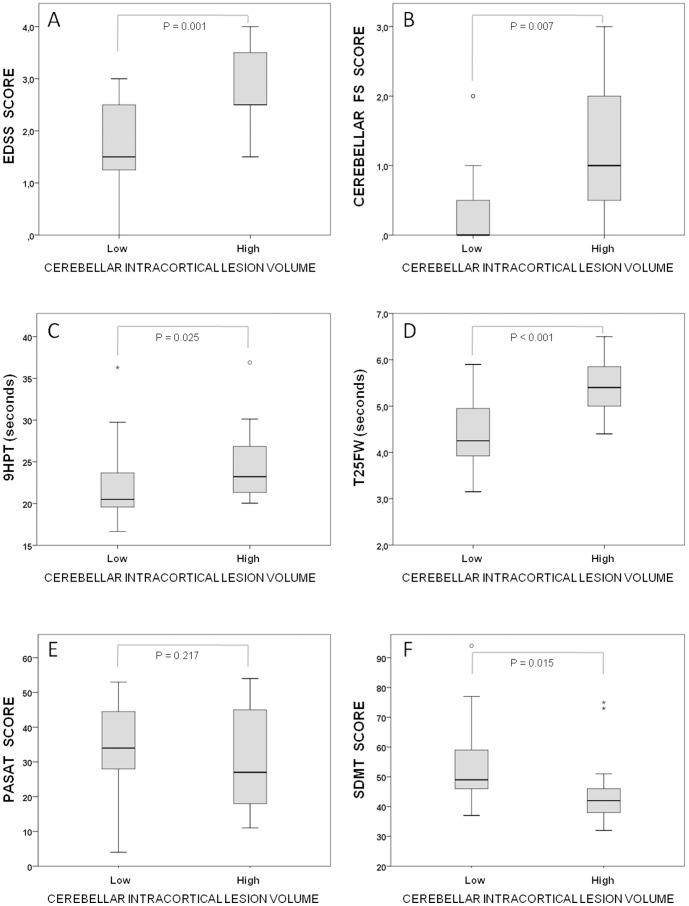
Burden of cerebellar intracortical lesions and clinical/cognitive outcomes. Box plots comparing patients with high and low burden (> 0.05 cm3 or<0.05 cm3) of cerebellar intracortical lesions on clinical (A – D) and cognitive outcomes (E, F).

Patients with high load of cerebellar leukocortical lesions had similar scores on clinical outcomes when compared to those with lower burden of these lesions. Conversely, patients with high burden of cerebellar intracortical lesions performed the T25FW and 9HPT tests at a slower pace (5.49 vs. 4.37 seconds, p<0.001; and 25.22 vs. 21.94 seconds, p = 0.025, respectively) and had higher scores on the cerebellar FS and total EDSS (1.25 vs. 0.33, p = 0.007; and 2.92 vs. 1.74, p = 0.001, respectively) (Figure 2).

On correlation analysis, cerebellar leukocortical lesions were significantly associated with cerebellar FS score while cerebellar intracortical lesions were related to all clinical outcomes (Table 2).

**Table 2:** Correlations between clinical and MRI factors and outcome measures.

Correlations		EDSS	Cerebellar FS	9HPT	T25FW	PASAT	SDMT
Disease duration	R	−0.046	0.220	−0.093	0.091	−0.123	0.053
	p	0.771	0.161	0.554	0.561	0.432	0.736
Age	R	−0.061	0.147	−0.016	**0.313**	0.143	−**0.247**
	p	0.697	0.353	0.895	**0.008**	0.230	**0.037**
Education	R	−0.153	0.078	−0.056	−0.172	**0.436**	**0.447**
	p	0.335	0.626	0.726	0.275	**0.004**	**0.003**
Cerebellar leukocortical Lesions	R	0.288	**0.322**	0.102	0.159	−**0.444**	−0.182
	p	0.072	**0.045**	0.532	0.328	**0.004**	0.261
Cerebellar intracortical lesions	R	**0.494**	**0.428**	**0.347**	**0.486**	−0.093	−0.260
	p	**0.001**	**0.007**	**0.028**	**0.001**	0.570	0.105
Brain cortical lesions	R	0.241	0.160	0.242	0.093	−**0.596**	−**0.450**
	p	0.134	0.330	0.133	0.568	**<0.001**	**0.004**
Brain WM lesions	R	0.191	**0.404**	0.231	0.064	−**0.667**	−**0.373**
	p	0.237	**0.010**	0.151	0.695	**<0.001**	**0.018**
Brain cortical volume	R	−0.070	−0.094	−0.075	−**0.297**	**0.378**	0.137
	p	0.665	0.553	0.538	**0.013**	**0.001**	0.259
Cerebellar GM volume	R	−0.179	−**0.357**	−0.125	−**0.318**	**0.303**	0.196
	p	0.251	**0.020**	0.302	**0.007**	**0.011**	0.103
Cerebellar WM volume	R	−0.293	−**0.336**	−0.229	−0.218	**0.378**	**0.251**
	p	0.056	**0.030**	0.057	0.069	**0.001**	**0.036**

Expressed are Spearman correlation coefficients (R) and respective p values. Significant associations are highlighted in bold.

9HPT: nine-hole peg test; EDSS: Expanded Disability Status Scale; FS: functional system; GM: grey-matter; NA: not applicable; PASAT: Paced Auditory Serial Addition Test; RRMS: relapsing-remitting multiple sclerosis; SDMT: Symbol Digit Modalities Test; T25FW: timed twenty-five foot walk test; WM: white-matter.

We performed partial correlations of cerebellar cortical lesions and EDSS controlling for brain cortical lesions and brain cortical volume (as covariates), and the correlation was still significant (R = 0.491, p = 0.002). This was also true for the FS cerebellar score (R = 0.463, p = 0.004).

After multivariate analysis, age, disease duration, years of education, and gender were not related to clinical outcomes, except for the T25FW test, where gender, disease duration and age were independent predictors (β = 0.75, 95% CI 0.27 to 1.23, p = 0.003; β = −0.05, 95% CI −0.095 to −0.007, p = 0.025; and β = 0.076, 95% CI 0.043 to 0.108, p<0.001, respectively).

High burden of cerebellar intracortical lesions was the only independent predictor for the EDSS (β = 1.109, 95% CI 0.575 to 1.642, p<0.001), cerebellar FS score (β = 0.904, 95% CI 0.349 to 1.459, p = 0.002), and to the 9HPT time (β = 3.107, 95% CI 0.011 to 6.203, p = 0.049).

For the T25FW time, high burden of cerebellar intracortical lesions and brain cortical volume remained as independent predictors but only the first was statistically significant (β = 1.062, 95% CI 0.607 to 1.518, p<0.001, and β = −0.005, 95% CI −0.009 to 0.000, p = 0.071). When those clinical factors found to be independent predictors of this test (age, disease duration and gender) were included in the model, cerebellar intracortical lesions still remained as an independent predictor (β = 0.965, 95% CI 0.59 to 1.34, p<0.001).

#### Cognitive

Patients with high burden of cerebellar leukocortical lesions had lower scores on the PASAT test (25.25 vs. 36.67, p = 0.013), while patients with greater volumes of cerebellar intracortical lesions had lower scores on the SDMT (43.25 vs. 53.18, p = 0.015) (Figure 2).

On correlation analyses, cerebellar leukocortical lesions were significantly associated with the PASAT score but cerebellar intracortical lesions were not related to cognitive outcomes (Table 2).

In multivariate analysis, number of years of education was the only clinical factor related to SDMT (β = 3.102, 95% CI 0.996 to 5.209, p = 0.005) and to PASAT score (β = 2.930, 95% CI 0.857 to 5.002, p = 0.007).

For the PASAT score, high burden of cerebellar leukocortical lesions (β = −6.066, 95% CI −12.296 to 0.165, p = 0.056), WML (β = −14.610, 95% CI −20.909 to −8.310, p<0.001), and brain cortical volume (β = 0.083, 95% CI 0.015 to 0.152, p = 0.018) remained in the model.

Both high burden of cerebellar intracortical lesions and brain WML showed a trend to predict SDMT score (β = −7.869, 95% CI −16.843 to 1.106, p = 0.084, and β = −7.837, 95% CI −16.371 to 0.698, p = 0.071, respectively).

### Cerebellar atrophy

There was a tendency to cerebellar intracortical lesions predict cerebellar GM volume (β = −8.55, 95% CI −17.74 to 0.65, p = 0.068). High burden of cerebellar leukocortical lesions was the only independent predictor to cerebellar WM volume (β = −3.65, 95% CI −6.93 to −0.37, p = 0.030).

## Discussion

This *in vivo* MRI study confirms a major role of cerebellar GM pathology in clinical disability of MS patients, and strengthens findings from previous research. [8] Cerebellar GM involvement in this disease has been clearly demonstrated in a number of neuropathological studies, concerning both cortical lesions and atrophy.[3] Alike the forebrain, similar types of cortical lesions are seen in the cerebellum, mainly extending over several folia.[1] However, pathological studies so far yielded little information about the clinical significance of cerebellar GM pathology and one study found no association between cerebellar cortical demyelination and clinical factors (i.e. age, gender and disease duration).[1]–[3]

MRI in-vivo visualization of cortical pathology provides a better opportunity to assess such clinical significance but lesions in the GM are mostly undetectable with traditional MRI sequences.[3] GM atrophy measurements can be done with 3D MRI acquisitions using different types of software. Therefore, this approach has been used by several studies which documented a progressive loss of brain parenchyma, starting at the earliest stages and continuing throughout the long course of the disease.[3] In particular for the cerebellum, using voxel-based morphometry, significant correlations were found between cerebellar volume estimates and clinical metrics as measured by 9HPT and EDSS cerebellar functional score.[6] On the other hand, Anderson *et al*, on a comprehensive evaluation of cerebellar damage using diffusion tractography and volumetric analysis, found that cerebellar WM volume was associated with 9HPT score in patients with primary progressive MS, independently of cerebellar GM volume.[16] We found significant correlations between cerebellar WM/GM volumes and clinical dysfunction (cerebellar FS) and information processing speed performance (PASAT) in patients with relapsing-remitting MS, supporting findings from recent research stressing the cerebellar role in cognitive functions and notably sequencing abilities.[4], [5] However, these associations with cerebellar volumes were not present after multivariate analysis, where cerebellar cortical lesions were more predictive.

Posterior fossa lesions are typical in MS but their visualization presents challenges for neuroimaging. Conventional MRI techniques can leave some infratentorial lesions undetected, and especially cerebellar GM lesions. Recently, a more sensitive MRI acquisition sequence, known as DIR, has become available and has been reported to identify significantly more inflammatory lesions in the infratentorial brain even compared with the T2 turbo spin-echo sequence. [3], [17] Although some studies have found significant associations between brain cortical lesions and cognitive dysfunction using this sequence, [3], [18] very few studies evaluated the clinical impact of cerebellar GM lesions. Calabrese *et al* evaluated the relative contribution of cerebellar cortical lesions in multivariate analysis and found them to be independent predictors of cerebellar disability. They also found cerebellar GM volume to be independent predictor of both cerebellar disability and EDSS score.[8] In accordance, we found that cerebellar intracortical lesions are predictors for both arm and leg dysfunction, and also for cerebellar and overall disability as measured by the EDSS, independently of brain cortical lesions or volume. As an example, high burden of cerebellar intracortical lesions increased T25FW time in one second, 9HPT time in three seconds and the EDSS score in one step.

We also aimed to discriminate the role of cerebellar intracortical and leukocortical lesions. Intracortical lesions were strongly related to clinical dysfunction and had a milder association with SDMT test performance, while leukocortical lesions were associated with the PASAT test score and cerebellar WM atrophy. Intracortical lesions represent around 50% of GM plaques in the cerebellum.[2] Some demyelinated plaques in the cerebellar cortex are also found in continuity with demyelination in the subcortical WM and sometimes associated with large WM plaques,[1] what may explain the contribution of cerebellar leukocortical lesions to cerebellar WM atrophy found in our study. Moreover, involvement of both GM and WM may disrupt important cerebello-cortical loops known to be involved in attention and other cognitive functions.[4], [5] Interestingly, Sastre-Garriga *et al* has recently shown with functional MRI an important cerebellar activation when PASAT test was used as paradigm.[19]

Although DIR MRI may improve detection of cortical lesions up to five times when compared with a conventional T2-weighted sequence, the vast majority are still missed by this technique, especially subpial lesions which may also have important contribution to clinical and cognitive dysfunction in MS patients.[3] Another disadvantage is its low signal-to-noise ratio, resulting in low agreement between observers. Therefore, some lesions considered as intracortical may in fact be leukocortical and vice-versa. Moreover, we have not included cerebellar WM lesions volumetry in our analysis and, therefore, we cannot discriminate if cerebellar leukocortical lesions influence is predominantly due to its GM or WM involvement. Future studies associating greater MRI field strengths and novel/improved sequences, such as 3D-DIR or phase sensitive inversion recovery may overcome this gap.[3], [20], [21]

In conclusion, cerebellar GM is widely affected in relapsing-remitting MS patients. Cerebellar GM pathology strongly contributes to clinical dysfunction in relapsing-remitting MS patients and also to information processing speed deficits in a lesser extent, independently of brain cortical lesions or volume. This damage can be monitored in-vivo with MRI. Further work is required to better characterize GM plaques and to assess its contribution to long term disability.
